# Gut microbiota and host genetics modulate the effect of diverse diet patterns on metabolic health

**DOI:** 10.3389/fnut.2022.896348

**Published:** 2022-08-18

**Authors:** M. Nazmul Huda, Anna C. Salvador, William T. Barrington, C. Anthony Gacasan, Edeline M. D'Souza, Laura Deus Ramirez, David W. Threadgill, Brian J. Bennett

**Affiliations:** ^1^Department of Nutrition, University of California, Davis, Davis, CA, United States; ^2^Obesity and Metabolism Research Unit, USDA, ARS, Western Human Nutrition Research Center, Davis, CA, United States; ^3^Department of Molecular and Cellular Medicine, Texas A&M Health Science Center, Texas A&M University, College Station, TX, United States; ^4^Department of Nutrition, Texas A&M University, College Station, TX, United States; ^5^Department of Veterinary Medicine and Biomedical Sciences, Texas A&M University, College Station, TX, United States; ^6^Leverhulme Quantum Biology Doctoral Training Centre, University of Surrey, Guildford, United Kingdom; ^7^School of Bioscience and Medicine, Faculty of Health and Medical Sciences, University of Surrey, Guildford, United Kingdom; ^8^Texas A&M Institute for Genome Sciences and Society, Texas A&M University, College Station, TX, United States; ^9^Department of Biochemistry and Biophysics, Texas A&M University, College Station, TX, United States

**Keywords:** gut microbiota, 16S, genetics, precision nutrition, metabolic health, glucose metabolism, insulin, body fat

## Abstract

Metabolic diseases are major public health issues worldwide and are responsible for disproportionately higher healthcare costs and increased complications of many diseases including SARS-CoV-2 infection. The Western Diet (WD) specifically is believed to be a major contributor to the global metabolic disease epidemic. In contrast, the Mediterranean diet (MeD), Ketogenic diet (KD), and Japanese diet (JD) are often considered beneficial for metabolic health. Yet, there is a growing appreciation that the effect of diet on metabolic health varies depending on several factors including host genetics. Additionally, poor metabolic health has also been attributed to altered gut microbial composition and/or function. To understand the complex relationship between host genetics, gut microbiota, and dietary patterns, we treated four widely used metabolically diverse inbred mouse strains (A/J, C57BL/6J, FVB/NJ, and NOD/ShiLtJ) with four human-relevant diets (MeD, JD, KD, WD), and a control mouse chow from 6 weeks to 30 weeks of age. We found that diet-induced alteration of gut microbiota (α-diversity, β-diversity, and abundance of several bacteria including *Bifidobacterium, Ruminococcus, Turicibacter, Faecalibaculum*, and *Akkermansia*) is significantly modified by host genetics. In addition, depending on the gut microbiota, the same diet could have different metabolic health effects. Our study also revealed that C57BL/6J mice are more susceptible to altered gut microbiota compared to other strains in this study indicating that host genetics is an important modulator of the diet-microbiota-metabolic health axis. Overall, our study demonstrated complex interactions between host genetics, gut microbiota, and diet on metabolic health; indicating the need to consider both host genetics and the gut microbiota in the development of new and more effective precision nutrition strategies to improve metabolic health.

## Introduction

Over the last decade, non-communicable diseases have increased dramatically due to changes in dietary patterns and lifestyles (1). Non-communicable diseases cause more than 80% of deaths in Western societies (2) with poor metabolic health being a major contributor. Metabolically unhealthy people have disproportionately high morbidity and mortality, and worse disease progression when challenged with infectious diseases like SARS-CoV-2 (3–5). The co-occurrence of several related risk factors (abdominal obesity, insulin resistance, hyperglycemia, hypertriglyceridemia, decreased HDL cholesterol, and hypertension) are summarized under the term “metabolic syndrome” (MetS). In the United States, nearly 35% of all adults and 55% of elderly adults aged 65 and over have MetS (6). People with MetS have 1.6 times higher health care costs compared to individuals free from any risk factors for the syndrome (7). MetS has become a global epidemic in part due to broad adoption of a Western diet (WD) (8), which is characterized by an excess of saturated fat, sodium, refined grains and sugar, and comparatively less vegetables, fruits, whole grains, dairy products and polyunsaturated fatty acids (PUFA) to other well-defined dietary patterns. WD induces metabolic diseases directly (9) and indirectly through altered gut microbiota (10).

Diet patterns (the quantity, variety, or combination of different foods and beverages in a diet) (11) vary across the globe, are influenced by culture (12), and are popularized in the media. Different diet patterns including the Mediterranean diet (MeD), Ketogenic diet (KD), and Japanese diet (JD) reflect broad differences in food and nutrient intake (13, 14). For example, the traditional MeD is characterized by higher intake of fruits, vegetables, whole-grain cereals, extra-virgin olive oil, nuts, and a lower intake of red meat and sweets. MeD is reported as a healthy diet that is associated with lower incidences of metabolic diseases compared to WD (14). KD contains high fat, moderate protein, and very low carbohydrate (15). KD treatment of obese individuals has shown to decrease body weight and body mass index, total cholesterol, LDL cholesterol, triglycerides, and blood glucose, and increase HDL cholesterol (16, 17). However, the effect of a KD on MetS still remains controversial (18, 19) and has raised concerns among some physicians due to its high-fat content (13). Moreover, little is known about the effect of KD on gut microbiota (19). The classic JD is rich in fish, seafood, and plant-based foods with minimal amounts of red meat, added sugars, and fat. The JD diet is considered healthy due to its association with improved metabolic health (20) and longer life expectancy (21). Though the JD has beneficial effects in a particular population, several studies failed to demonstrate similar benefits in other populations. Host genetics and gut microbiota may explain the interindividual variation in the response to different dietary patterns and susceptibility to diet-induced metabolic diseases.

We have previously reported (22) that the diet effect on host metabolic health varies depending on the genetic background of the mouse strain. In brief, we found that the WD induced adiposity in all mice, which was significantly more pronounced in C57BL/6J compared to other mouse strains. Elevated fasting blood glucose and poor glucose tolerance (GTT-AUC) were observed in C57BL/6J and FVB/NJ mice consuming a WD diet pattern, which was observed minimally in A/J mice, and was not observed in NOD/ShiLtJ mice. KD prevented increased adiposity in C57BL/6J and A/J mice but did not have any effect in FVB/NJ or NOD/ShiLtJ mice. Additionally, KD induced poor glucose tolerance in NOD/ShiLtJ mice that was not observed in any other mice strains. The JD improved glucose tolerance in C57BL/6J and FVB/NJ but did not have an effect in any other mouse strains. JD also decreased body fat percentage in A/J, C57BL/6J, and FVB/NJ but did not affect NOD/ShiLtJ mice. On the other hand, MeD improved glucose tolerance in C57BL/6J mice. Importantly, we found food intake was poorly correlated with fat gain across all diets, further supporting the importance of integrating gut microbiota and host genetics to understand the diet effect on metabolic health.

The diversity and composition of the gut bacteria have been intensely studied, as well as their impact on metabolic health (23–27). Studies suggest that gut microbiota can modulate MetS (27–29). Conversely, risk factors of MetS also affect the gut microbiota and further worsen metabolic health (30). In many cases, diet is the major driving force for modulating the gut microbiota (31–34). Diet effects on clinically relevant phenotypes can vary according to an individual's microbiota and genetic variation (35). Thus relationships between gut microbiota, diet, and host genetics are exceedingly complex (27). A recent study (36) with a large European cohort demonstrated the interactions between genetic variation, diet, and gut microbiota. For example, lactose intolerant individuals (defined as not having a functional LCT-MCM6 locus: rs4988235) who consumed dairy products regularly had increased *Bifidobacterium* abundance compared to lactose-intolerant individuals who did not consume dairy regularly. Similarly, *Faecalicatena lactaris* levels were increased in non-A/B/AB-secretors (inactivated FUT2 gene: rs601338) individuals who consumed a high-fiber diet. These data further suggest that the complex interactions of diet, genetics, and microbial abundance and function have important effects on biological phenotypes and ultimately disease risk. Ascertaining these complex gene-diet-microbiota associations can improve our understanding of dietary needs as we move toward precision nutrition. Given the complexity of the interactions and diversity among population in terms of genetics and diet; more work remains to fully understand the relationship between gut microbiota, diet, and host genetics.

In this study, we evaluated the effect of four popular diet patterns (MeD, KD, JD, WD) and a mouse control chow in four metabolically diverse inbred mouse strains (C57BL/6J, A/J, FVB/NJ, and NOD/ShiLtJ). C57BL/6J mice are the most widely utilized laboratory mouse strain as the metabolic abnormalities of the C57BL/6J mouse closely parallel that of the human obesity progression pattern (37, 38). In contrast, A/J mice are genetically resistant to developing diet-induced metabolic diseases (38, 39), despite having low levels of activity. FVB/NJ is a physically active mouse strain (40, 41) and is also resistant to diet-induced metabolic diseases (42). NOD/ShiLtJ, a popular Type I diabetic mouse model, is prone to develop spontaneous insulin deficiency due to pancreatic beta-cell destruction by autoimmunity (37). Here, we report the microbiome aspect of the study. We found that depending on the underlying host genetics, diet modulates gut microbiota differently. Moreover, we found that diet, gut microbiota, and host genetics modulate each other's effects on host metabolic health.

## Methods

### Study design and sample collection

Detailed study design and study procedures have been reported previously (22). In brief, four-week-old A/J, C57BL/6J, FVB/NJ, and NOD/ShiLtJ mice (The Jackson Laboratory, Bar Harbor, ME) were acclimated for two weeks and then randomized to one of five diets: traditional Mediterranean diet (MeD), Japanese diet (JD), typical American diet (mentioned as Western diet (WD) hereafter), ketogenic diet (KD), or control mouse chow with five mice per diet, sex, and strain (Supplementary Figure 1). Mice were housed five per cage and maintained at 22°C under a 12-h light cycle. The mice were maintained on the experimental human and mouse control diets from 6 to 30 weeks of age. Fecal samples were collected at 30 weeks of age and stored at −80°C until further processing. The final dataset had 16S rRNA gene amplicon sequencing data from 149 mice (*n* = 27–34 per diet, 27–44 per strain, and 3–10 per diet-strain, Supplementary Table 1). The study protocol was approved by Texas A&M University Institution Animal Care and Use Committee (IACUC) protocol number 2017-0076. All experiments were performed in accordance with relevant guidelines and regulations.

### Diet

MeD, JD, WD, and KD diets were designed to match human diets as closely as possible in terms of macronutrient ratio, fiber content, types of ingredients, and fatty acid ratios to the human diets according to the Food and Agriculture Organization's Food Balance Sheets from Greece and Japan in 1961 (43), Department of Agriculture's 2008 Dietary Assessment of Major Food Trends (44), and diet consumed by the Masaai population (45). Detailed diet formulation, and macro- and micro-nutrient composition have been previously reported (22). The five experimental diets were assigned randomly to each of the four inbred mouse strains separately.

### Clinical phenotype assays

Blood samples were collected for clinical phenotyping following a 6h fasting. Insulin concentrations were quantified using a Mouse Serum Adipokine Immunoassay ELISA kit (Millipore, Bedford, MA). A glucose tolerance test (GTT) was carried out after 6h of fasting. Blood glucose levels were measured with a Bionome GM100 glucose monitor (Bionome USA). Detailed GTT methodology has been reported earlier (22).

### Anthropometric phenotype assays

Body composition was measured by EchoMRI-130 Body Composition Analyzer, which has been detailed earlier (22).

### Gut microbiota assay

Stool DNA was extracted by using Fast DNA Spin Kit for Soil (MP Biomedicals, Solon, OH), according to the manufacturer's instructions. A mixed template amplicon library of the hypervariable region V3-V4 of the bacterial 16S rRNA gene was prepared by using 341F and 805R primer set (46). Amplicon PCR was carried out by KAPA HiFi HotStart ReadyMix (Roche, Basel, Switzerland) and the product was cleaned using AMPure XP beads (Beckman Culture, IN, USA). A second PCR was performed to attach 8 nucleotide dual indices and Illumina sequencing adapters using the Nextera XT (Illumina, CA, USA). The amplicon library was sequenced using the Illumina MiSeq platform. Sequences were de-multiplexed and amplicon sequence variants (ASV) was determined using the open-source software QIIME2-DADA2 pipeline (47). Taxonomy was assigned using the SILVA 132 reference database (48) customized for QIIME2 for 16s V3-V4 (341F/805R) region of sequences at the threshold of 99% pairwise identity. A total of 11,766,771 sequences remained after quality filtering and demultiplexing with an average of 54,476 ± 11,616 (mean ± SD) sequences per sample for ASV picking. After removing noised and chimeric sequences, a total of 7,084,823 sequences with an average 32,800 ± 7,148 per sample were in the final ASV table. The final dataset had 16S rRNA gene amplicon sequencing data from 176 mice (*n* = 27–34 per diet).

### Statistical analysis

Statistical analyses were performed using R version V4.0.3 for Windows (49). Metadata continuous variables were analyzed for Normality using the Shapiro-Wilk Normality Test and QQ-normal plot. Variables with the Shapiro-Wilk “W” value ≥ 0.95 were considered Normal. Non-Normally distributed metadata variables were transformed by natural log, square root, square, or Box-Cox power transformation. If no appropriate transformation was found, the variables were normalized rank transformed. The ASV table was filtered by removing any ASV present in fewer than 5 samples and with a relative abundance ≤ 0.005% across all samples to calculate the differential taxa abundance. We utilized ANCOM-2.1 (50) with adjusted for confounding factors (e.g., sex, mouse strains, and diet) to determine the differential ASV and genera abundance as described in the results below. Shannon diversity index on the alpha rarefaction curve reached a plateau at about 3,000 sequences per sample. We performed a single rarefaction at a sequence depth of 15,000 sequences per sample. α-diversity (Shannon diversity index, Faith's phylogenetic diversity, and observed ASV) and β-diversity (unweighted UniFrac, weighted UniFrac, and Bray Curtis) were calculated from the unfiltered ASV table. Differences in microbial community β-diversity were tested by ADONIS using 999 permutations in the R package Vegan (51) upon controlling for the confounding factors as described in the result below. Principal coordinate (PCoA) analysis was carried out by PhyloSeq (52). ANOVA analysis was performed to compare between groups adjusted for confounding factors. Diet, strain, and microbial diversity together showed increased variance inflation factor (VIF) and therefore separate ANOVA models had been developed for analyzing the interactions between diet and diversity, and mouse strain and diversity. Graphs were prepared by GGplot2 (53).

## Results

### Diet dominates host genetics in shaping gut microbiota

**Figure 1:**
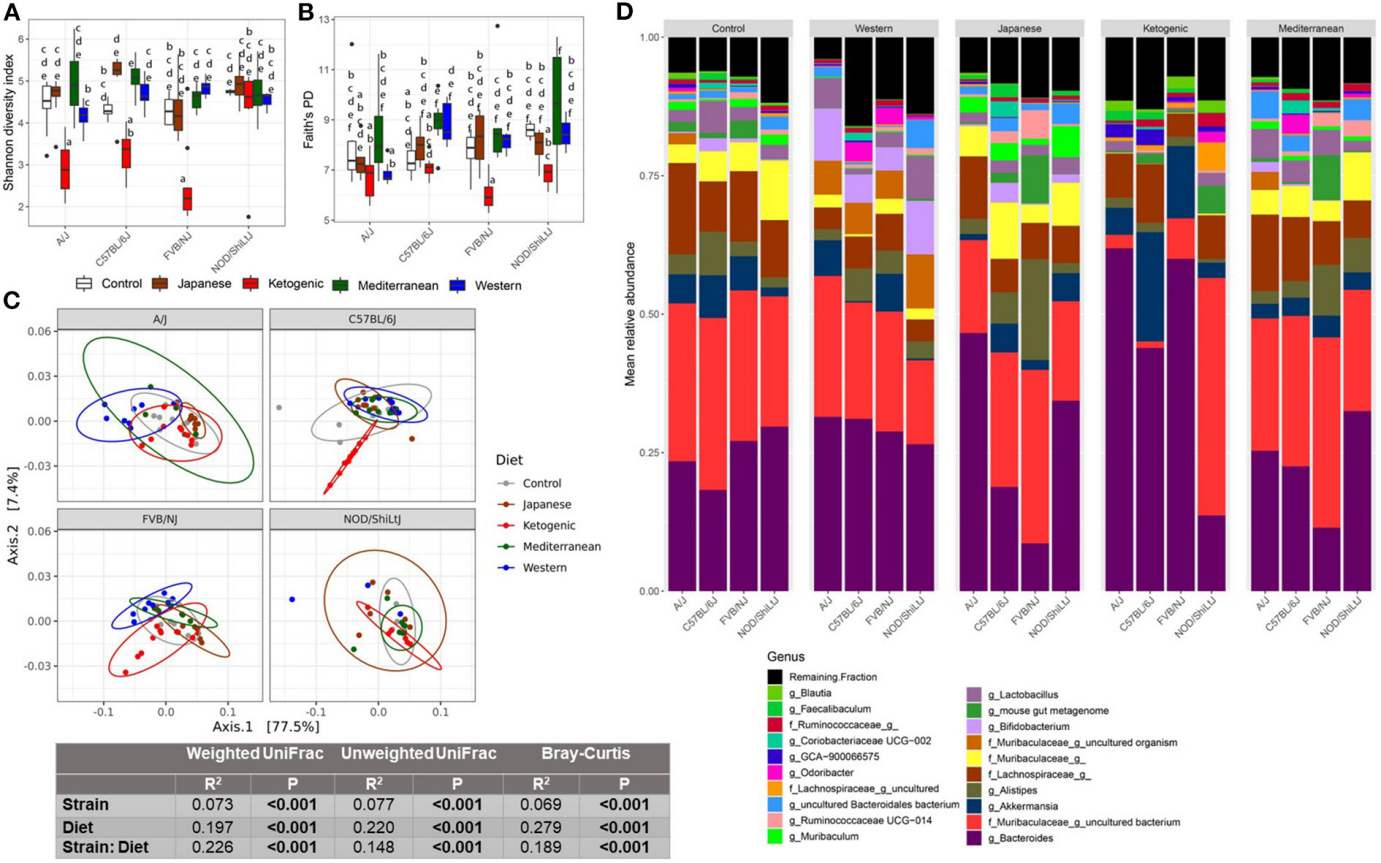
Diet-gene interaction on gut microbiota. Shannon diversity index **(A)** and Faith's PD **(B)** α-diversity by diet and strain. Boxes without a common letter are significantly (*p* < 0.05) different from others. Boxes with common letters represent no significant difference. 2-way ANOVA with Tukey's *post hoc* analysis was performed to determine the statistical differences. **(C)** Weighted UniFrac β-diversity PCoA plot. The color of the point represents diet. The ellipse on the principal coordinate analysis plot indicates 95% CI of the clusters by groups. Inset box is the results of ADONIS test on diet, strain, and their interaction. ADONIS analyses were adjusted for sex as a covariate. **(D)** Mean relative abundance of top 20 genera in A/J, C57BL/6J, FVB/NJ, and NOD/ShiLtJ mice treated with control mouse diet, WD, JD, KD, or MeD.

This study aimed to determine how popular dietary patterns modulate gut microbial diversity and composition in genetically diverse mouse strains and how the gut microbiota interacts with diet and host genetics to alter metabolic health. We assayed gut microbiota following 24 weeks of diet treatment using the widely used 16S rRNA gene amplicon sequencing method. As expected, gut microbial diversity and composition were significantly influenced by both diet and genetics (Figures 1A–D). In particular, the microbial α-diversity was significantly different among the diets (Supplementary Figures 2A–C) with a higher diversity observed in MeD and a lower diversity in KD compared to WD and mouse control chow. Here we report three different α-diversity measures; observed ASV, Shannon diversity index, and Faith's Phylogenetic Diversity (Faith's PD). The observed ASV (commonly known as observed species) depends on the number of different ASV present in a sample that represents the richness of the sample. Shannon diversity index represents the richness of the sample weighted by the abundance of each of the ASV, while Faith's PD is the phylogenetic richness of the sample, an important diversity measure that considers the similarity between bacteria based on shared evolution. In our study, we found that diet explains 47, 36, 37% variability of the Shannon diversity index, Faith's PD, and Observer ASV, respectively. In contrast, the genetic background of the mouse strain explains only 8, 8, and 5% variability of the Shannon diversity index, Faith's PD, and Observer ASV, respectively.

In addition to α-diversity measures, we presented three commonly used β-diversity (Bray-Curtis, unweighted UniFrac, and weighted UniFrac) measures. β-diversity is a measure of how similar or dissimilar two or more microbial communities are. Bray-Curtis is a quantitative, non-phylogenetic β-diversity metric that considers only the abundance of the features. Unweighted UniFrac is a qualitative, phylogenetic β-diversity metric that depends on only the presence or absence of features and the phylogenic distance of the features (54). Unweighted UniFrac is more sensitive to rare bacterial taxa. Weighted UniFrac is a qualitative, phylogenetic β-diversity metric that additionally adjusts the unweighted UniFrac measures with the abundance of the features (54). Both weighted UniFrac and Bray-Curtis consider the relative abundance of the ASV and are more sensitive to the most abundant taxa. However, Bray-Curtis considers all features are equally dissimilar, but weighted UniFrac accounts for similarity based on shared evolution. All above mentioned β-diversity measures were significantly different among the diets and mouse strains (Figure 1C; Supplementary Figures 3B,C, 4). For β-diversity, diet explained 20% to 28% variability of the gut microbiota, whereas genetics explained only 6–8% variability in β-diversity. These data highlight the dramatic effects diet can have on the gut microbiota as compared to host genetics (mouse strain in this case).

### Host genetics modulate the association between diet and gut microbiota

The diet-induced alteration of the gut microbiota was influenced by the genetics of the host. In particular, a significant diet ^*^ genetic interaction was observed for both α and β-diversity measures. For instance, the KD significantly decreased the α-diversity measures (specifically Shannon diversity index, observed ASV, and Faith's PD) in FVB/NJ mice compared to both WD and mouse chow (Figures 1A,B; Supplementary Figure 3A). However, the effects of the KD on α-diversity measures were muted (limited to Shannon diversity index) in A/J mice, and absent in NOD/ShiLtJ mice.

Consistent with α-diversity, significant diet ^*^ genetic interactions on all microbial β-diversity measures were also observed (Figure 1C; Supplementary Figures 3B,C). Therefore, we determined the diet effect on the gut microbiota for each individual mouse strain using ADONIS function in the R package vegan and found that depending on the genetic background, the diet altered β-diversity measures differently with a range of 34–59%, 33–48%, and 45–57%, respectively, for weighted UniFrac, unweighted UniFrac, and Bray-Curtis β-diversity measures (Supplementary Table 2). These results demonstrate that genetics influences diet-induced alteration of microbial composition. The community differences among diets and strains were also reflective of large alterations in the mean relative abundance of the specific bacterial genera (Figure 1D) and ASVs (Supplementary Figure 3D).

**Figure 2:**
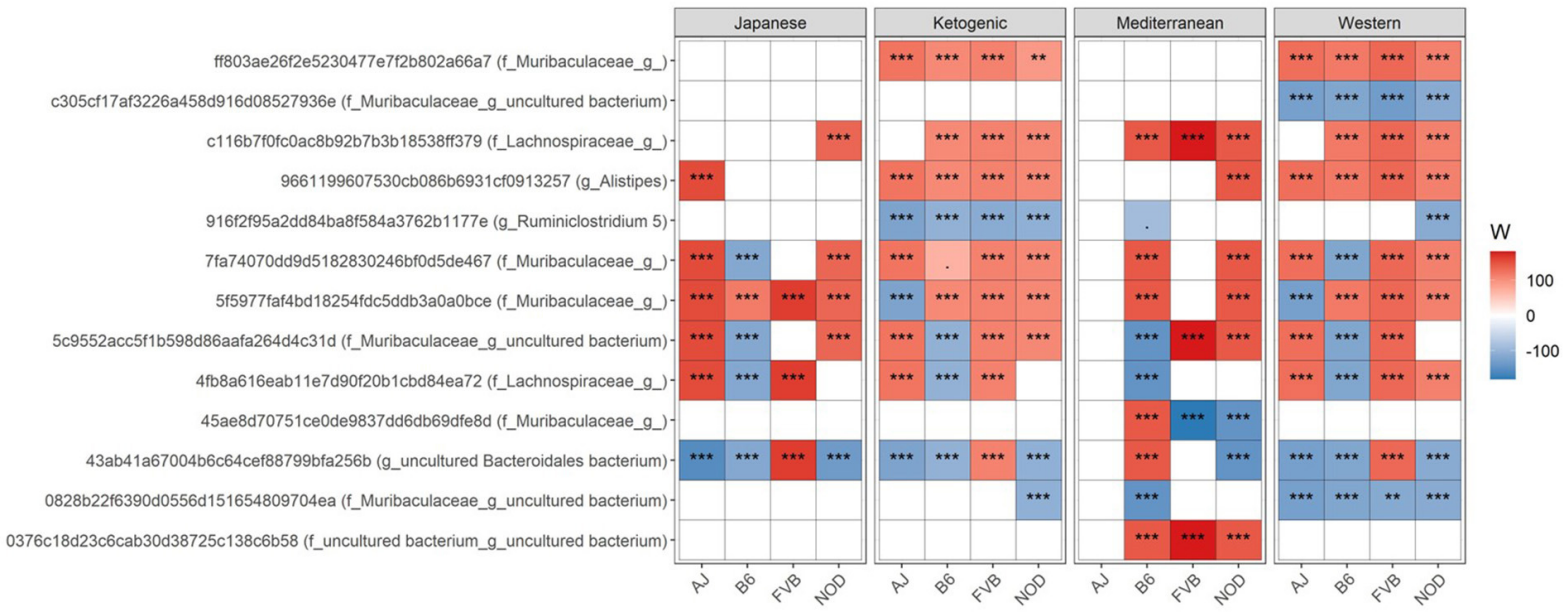
Differential ASV abundance between controlled mouse diet and four popular human diet patterns. The top 20 most differentially abundant (based on cumulative ANCOM W-value) ASV were selected for the graph. On the Y-axis, maximum taxonomic information has been presented with the ASV ID. For easier presentation, ANCOM W values were converted to negative if the CLR mean abundance is lower in the popular diet patterns compared to control mouse diet. Red indicates higher ASV abundance in the popular diet pattern and blue represents higher ASV abundance in the control diet group. White represents a non-significant result obtained from ANCOM analysis. ANCOM models were FDR (BH-method) corrected and a significant sub-hypothesis test at a level of adj.*P* < 0.05 was counted toward W value. Models were adjusted for sex as a confounding factor and were stratified by mouse strains. “***” = ≥W_0.9_, “**” = ≥W_0.8_, and “.” = ≥W_0.6_. The full list of the differential ASV abundance is available in Supplementary Table 3. Corresponding differential genera abundance has been depicted in Supplementary Figure 5, and the full list is available in Supplementary Table 4.

We then focused on which specific bacteria are differentially abundant between mouse control chow and popular human diets. Several bacterial ASVs (Figure 2; Supplementary Table 3) and genera (Supplementary Figure 5; Supplementary Table 4) were found enriched or depleted in popular human diet patterns compared to control mouse chow. In general, the relative abundance of bacterial ASVs belong to family *Muribaculaceae, Lachnospiraceae*, and genus *Alistipes* were consistently increased in human popular diets compared to mouse control chow (Figure 2), whereas *Ruminiclostridium* abundance was lower in human popular diets compared to the mouse control chow. Similarly, a genus under the family *Muribaculaceae* was enriched; and *Ruminococcaceae UCG-005, Rombutisa*, and *Parabacteroides* were depleted in human popular diets compared to control mouse chow (Supplementary Figure 5), indicating that mouse chow typically used in pre-clinical studies has a differential effect on gut microbiota compared to human-relevant diets and could alter interpretations of metabolic studies when only considering mouse chows.

Then to understand how WD contributes to higher metabolic diseases compared to other human diets used in this study, we focused on the differential bacteria abundance in MeD, KD, or JD diet patterns compared to WD. We found several differences in gut genera abundance in MeD, KD, and JD compared to the WD (Supplementary Table 5; Supplementary Figure 6). For instance, we found that relative abundance of short-chain fatty acids (SCFA) producing bacteria *Faecalibaculum, Blautia*, and *Lactococcus* were higher in KD, JD and MeD compared to WD. However, *Ruminococcaceae UCG-005* abundance was lower in JD, KD, and MeD compared to WD. Unexpectedly, we found the abundance of *Bifidobacterium* was higher in WD compared to other diets in this study. However, as we discuss below, we cannot determine which species or subspecies of the *Bifidobacterium* are enriched in WD using a 16S rRNA gene amplicon sequencing. Other notable bacterial genera that were differentially abundant compared to WD were *Akkermansia, Butyricicoccus, Anaeroplasma, Desulfovibrio, Lachnospiraceae, Odoribacter, Rikenella, Citrobacter, Dubosiella, Faecalibaculum, Roseburia, Ruminiclostridium 9, Streptococcus, Turicibacter, Muribaculum*, and *Parabacteroides*, which varied among diets and mouse strains (Supplementary Table 5; Supplementary Figure 3D). These results indicate that the differential gut microbial abundance due to diet treatment is significantly influenced by background genetics. For example, only in NOD/ShiLtJ mice, *Roseburia* was significantly decreased in KD and JD while being increased in MeD (Supplementary Table 5). The differential ASV abundance between diets also varied significantly depending on the mouse strain (Supplementary Table 6). For instance, an ASV belongs to the genus *Akkermansia* was >4-fold higher only in C57BL/6J mice fed KD as compared to those fed a WD (Supplementary Table 6). This result indicates that depending on the background genetics of the host, the same diet may show significant heterogeneity in its effects on the enrichment or depletion of specific gut bacteria.

### Diet and gut microbiota interact to affect host body composition

**Figure 3:**
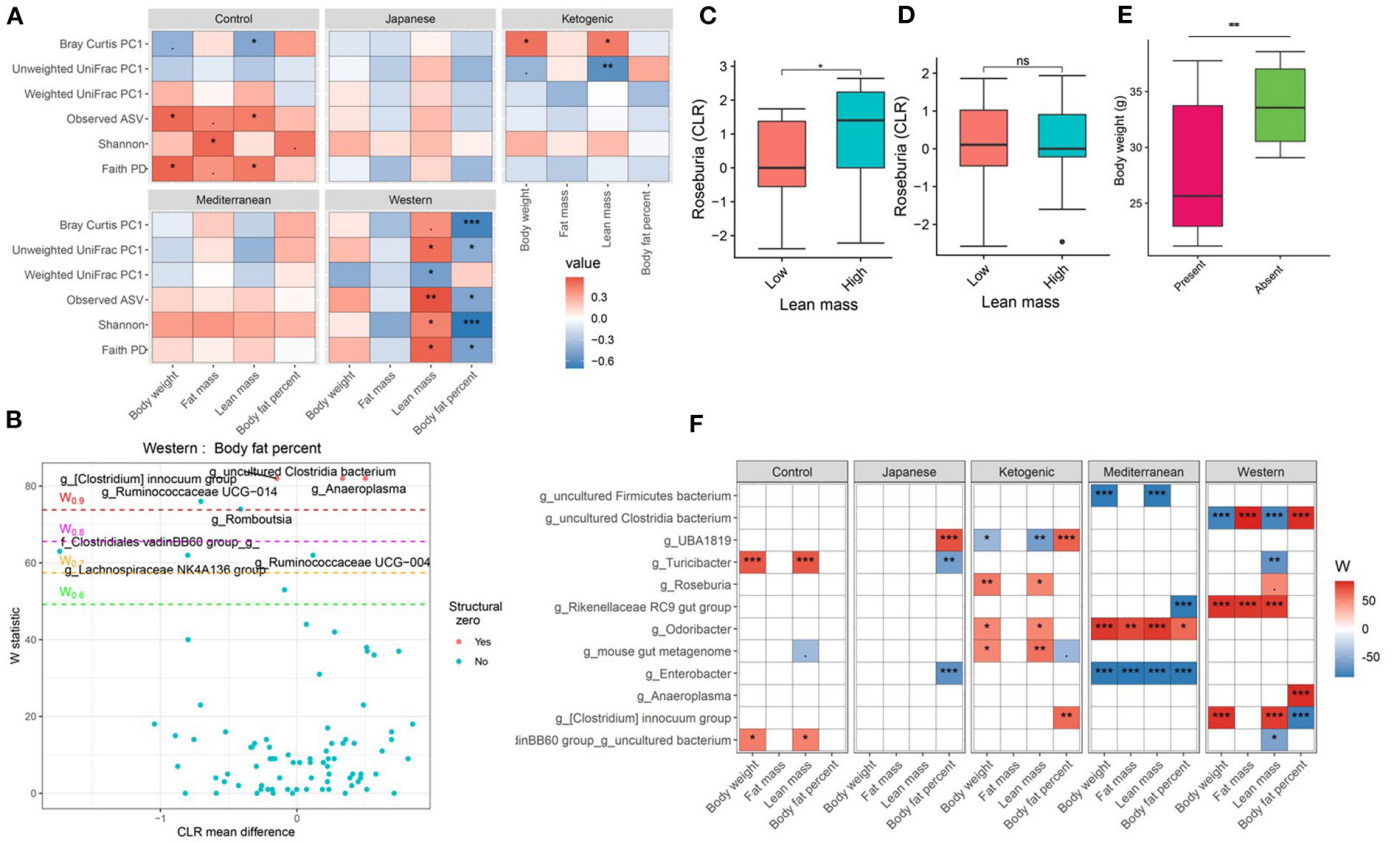
Diet and gut microbiota interact to alter host body composition. **(A)** Association between body composition measures and α-diversity or first principal component (PC1) of β-diversity measured for each diet determined by using Spearman correlation. As indicated, red represents a positive correlation and blue represents a negative correlation. *P*-values were adjusted for multiple comparisons using BH method. “***” = adj.*p* < 0.001, “**” = adj.*p* < 0.01, “*” = adj.*p* < 0.05, and “.” = adj.*p* < 0.10. **(B)** Volcano plot of the differential genera abundance between high (above median) and low (below median) body fat percentage in mice maintained on western diet determined by ANCOM analysis. Structural zero represents the presence of the bacteria in one group and complete absence in another group. **(C)** Relative abundance of the *Roseburia* in mice having high and low lean mass maintained on the ketogenic diet. **(D)** Relative abundance of the *Roseburia* in mice with high and low lean mass maintained on the Mediterranean diet. **(E)** Bodyweight distribution of mice maintained on the Mediterranean diet by the presence or absence of *Enterobacter* in their gut microbiome. Mean abundance was compared using the Wilcoxon rank-sum test. For **(C–E)** “**” = *P* < 0.01, “*” = *P* < 0.05, and “ns” = not significant. **(F)** Differential genera abundance between high (above median) and low (below median) body composition measures determined by ANCOM analysis. 20 most differentially abundant (based on cumulative ANCOM W-value) were selected for the graph. For easier presentation, ANCOM W values were converted to negative if the CLR effect size is negative. Red indicates higher genera abundance in the above-median group and blue bacteria represents higher genera abundance in the below-median group. White represents a non-significant result obtained from ANCOM analysis. Models were adjusted for mouse strain and sex as confounding factors. The median was calculated for each diet. ANCOM models were FDR (BH-method) corrected for multiple comparisons and a significant sub-hypothesis test at a level of adj.*P* < 0.05 was counted toward W value. For **(F)**, “***” = ≥W_0.9_, “**” = ≥W_0.8_, “*” = ≥W_0.7_, and “.” = ≥W_0.6_.

The gut microbiota is critical to digestion and thus variation in the gut microbiome can affect availability of energy substrates, host metabolism, and body composition. Therefore, we next focused on the association between gut microbiota and host body composition between diet groups. In the WD, all three microbial α-diversity measures were positively correlated with lean mass and negatively associated with body fat percentage (Figure 3A), indicating high microbial diversity is associated with better metabolic health. However, α-diversity measures in the MeD, KD, and JD did not show any significant association with body composition, and Faith's PD and observed ASV showed a positive correlation with body weight in controlled mouse chow. This result suggests a significant interaction between diet and α-diversity measures on body composition phenotypes. For example, Shannon diversity index explains 21, 0.3, 0.1, 7, and 50% variance of the body fat percentage respectively for control mouse chow, JD, KD, MeD, and WD, indicating a WD induces profound alteration in gut microbiome and dramatically affects body fat percentage (Table 1). The interaction between Shannon diversity index and diet on body fat percentage remained significant after adjusting for sex as a covariate (Supplementary Table 7).

**Table 1:** Percent phenotypic variance explained by gut microbial alpha and beta diversity measures in each of the experimental diet.

	**Faith's PD**	**Observed ASV**	**Shannon**	**Weighted UniFrac PC1**	**Unweighted UniFrac PC1**	**Bray Curtis PC1**
**Control**
Body weight	27.4%	26.1%	4.7%	6.5%	6.6%	15.7%
Fat mass	13.8%	16.7%	26.4%	0.1%	1.1%	1.3%
Lean mass	20.8%	19.9%	1.1%	7.0%	7.6%	20.9%
Body fat percentage	2.9%	5.6%	21.4%	2.2%	1.5%	11.4%
Fasting glucose	0.2%	1.5%	4.2%	3.3%	0.0%	3.0%
Insulin	0.4%	0.3%	2.1%	15.7%	0.2%	19.7%
GTT	15.7%	11.3%	0.1%	2.0%	0.6%	6.8%
**Japanese**
Body weight	0.5%	0.8%	7.2%	0.7%	0.2%	1.6%
Fat mass	11.4%	2.2%	1.2%	2.9%	6.2%	2.8%
Lean mass	1.9%	5.5%	5.3%	2.0%	4.9%	0.2%
Body fat percentage	12.8%	4.4%	0.3%	4.8%	14.4%	4.2%
Fasting glucose	0.0%	0.2%	0.4%	2.1%	10.5%	11.1%
Insulin	0.1%	0.7%	4.4%	2.1%	22.3%	1.9%
GTT	0.4%	4.2%	6.4%	5.5%	23.4%	9.9%
**Ketogenic**
Body weight	2.3%	0.8%	7.2%	2.1%	14.4%	23.1%
Fat mass	6.6%	3.5%	0.7%	14.1%	0.5%	1.0%
Lean mass	0.8%	0.1%	5.4%	0.0%	34.0%	20.2%
Body fat percentage	3.0%	2.3%	0.1%	11.1%	8.8%	1.0%
Fasting glucose	2.6%	1.2%	11.6%	16.8%	0.4%	31.3%
Insulin	17.0%	16.6%	0.3%	0.1%	1.8%	13.7%
GTT	1.7%	0.4%	12.0%	11.8%	0.3%	27.8%
**Mediterranean**
Body weight	1.7%	2.2%	11.4%	2.2%	3.9%	0.8%
Fat mass	0.3%	0.6%	13.4%	0.0%	0.9%	3.3%
Lean mass	2.8%	2.9%	9.5%	4.8%	14.7%	5.3%
Body fat percentage	0.1%	0.1%	7.0%	0.7%	6.6%	7.7%
Fasting glucose	1.2%	2.9%	22.1%	12.4%	18.3%	23.2%
Insulin	9.5%	4.4%	1.4%	3.8%	11.3%	31.3%
GTT	3.5%	0.3%	12.0%	5.5%	0.4%	0.1%
**Western**
Body weight	6.5%	10.7%	0.6%	17.7%	5.5%	0.6%
Fat mass	2.9%	1.7%	19.3%	3.7%	1.7%	11.3%
Lean mass	27.8%	33.3%	18.5%	24.2%	24.4%	14.4%
Body fat percentage	23.5%	21.1%	49.5%	2.7%	18.0%	42.1%
Fasting glucose	36.1%	38.5%	7.2%	31.5%	42.9%	10.8%
Insulin	0.5%	2.5%	0.2%	40.0%	0.2%	0.0%
GTT	36.9%	46.7%	18.1%	28.3%	25.8%	11.4%

Percent variance explained was calculated from the correlation coefficient.

Next, we focused on the association between microbial β-diversity and body composition to understand the effect of the overall microbial composition. Since principal component 1 (PC1) explains most of the variability of multidimensional data (55), we determined the correlation between PC1 of β-diversity measures and clinical phenotypes and body composition phenotypes. This approach was previously used in the microbiome field to demonstrate the association between microbial β-diversity and a higher number of clinical or metabolic phenotypes (56–58). Similar to measures of α-diversity, we observed that the WD has the most pronounced association between microbial β-diversity and body composition compared to other diets in the study (Figure 3A). The first principal component of Bray-Curtis β-diversity explains 11, 4, 1, 8, and 42% variance of the body fat percentage respectively for control mouse chow, JD, KD, MeD, and WD, indicating the changes in the microbial abundance due to diet treatment may explain the differential adipogenicity among diets (Table 1). The interaction between Bray-Curtis PC1 and diet on body fat percentage remained significant even after adjusting for sex as a covariate in the ANOVA model (Supplementary Table 8).

We then determined if any specific gut bacterial ASVs or genera are associated with body composition and found several gut bacteria were associated although the associations varied depending on diet composition (Supplementary Tables 9, 10). For instance, with WD, mice having lower body fat percentage were enriched with several bacterial genera including *Ruminococcaceae UCG-014, Lachnospiraceae NK4A136 group, Turicibacter, Ruminococcus 1*, and a genus under the family Clostridiales vadinBB60 group compared to mice having higher body fat percentage after adjusting for the mouse strain and sex as covariates (Figure 3B). With KD, mice having a higher body fat percentage were associated with 1.4-fold higher enrichment of *UBA1819*, a genus under the family Ruminococcaceae compared to those having a lower body fat percentage (Figure 3F; Supplementary Table 9). The same bacteria *UBA1819* was found about 0.8-fold lower in mice with higher lean mass, and 0.6-fold lower in higher body weight mice indicating a consistent association with body composition. Similarly, an uncultured bacterium under family *Clostridia* was positively associated with body fat percentage and negatively associated with the lean mass with WD. On the other hand, *Odoribacter* was positively associated with higher body fat percentage (1.5-fold enriched), body weight (0.8-fold lower), and lean mass (0.8-fold lower) with MeD. *Roseburia* was found positively associated with lean mass with KD (Figure 3C) but not with MeD (Figure 3D). Conversely, the presence of *Enterobacter*, a bacterium under Proteobacteria phylum was associated with mice with lower body weight, fat mass, and lean mass, which was completely absent in mice with higher body weight, fat mass, and lean mass consuming MeD (Figure 3E; Supplementary Table 9). The differential ASV abundance between high and low body composition phenotypes also varied depending on the dietary patterns (Supplementary Table 10). These study results indicate that gut microbiota is associated with host body composition and the association between gut microbiota and host body composition may vary in different diet patterns.

### Diet and gut microbiota interact to affect host glucose metabolism

**Figure 4:**
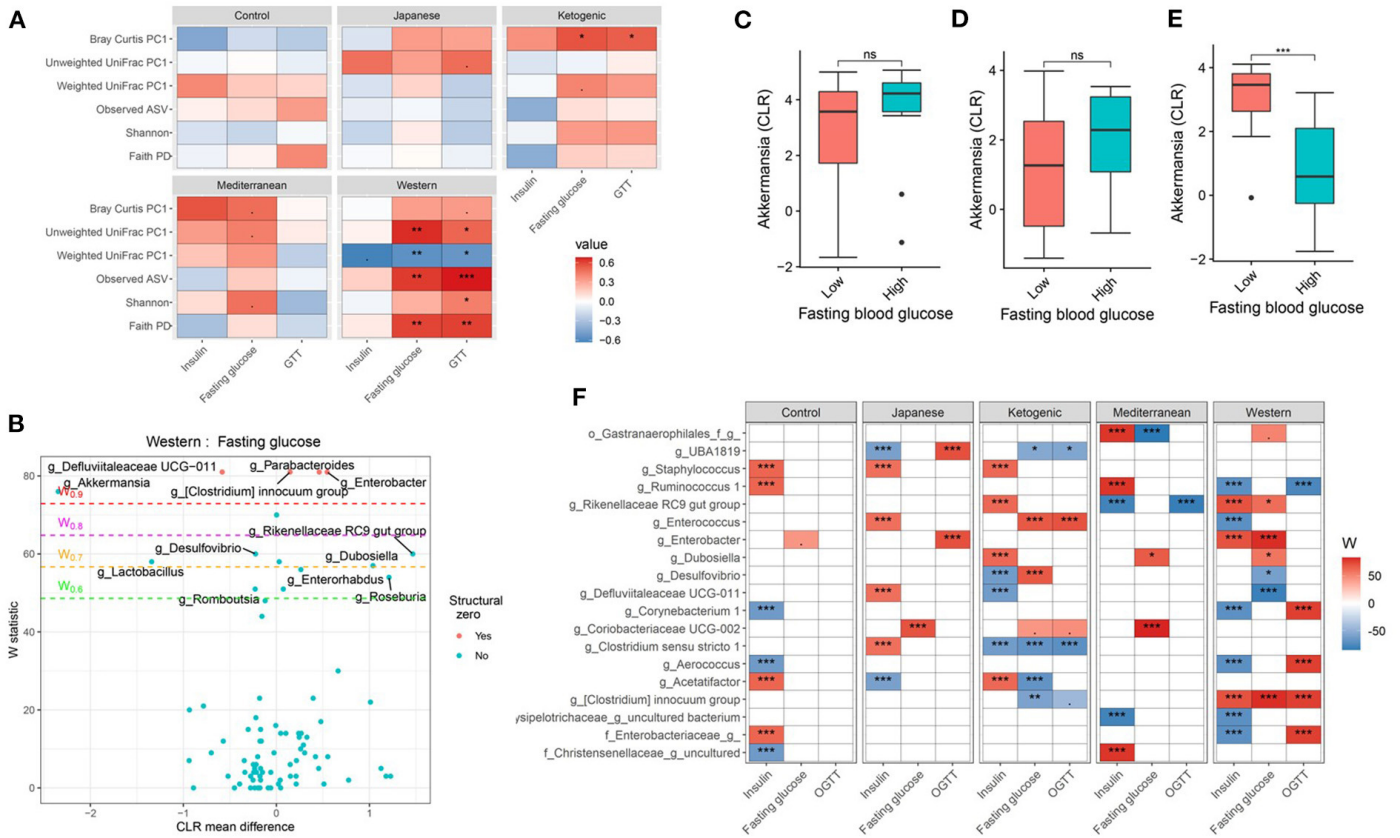
Diet and gut microbiota interact to modulate host's glucose metabolism. **(A)** Association between fasting blood glucose parameters and α-diversity or first principal component (PC1) of β-diversity measured for each diet determined by using Spearman correlation. As indicated, red represents a positive correlation and blue represents a negative correlation. P-values were adjusted for multiple comparisons using BH method. “***” = adj.*p* < 0.001, “**” = adj.*p* < 0.01, “*” = adj.*p* < 0.05, and “.” = adj.*p* < 0.10. **(B)** Volcano plot of the differential genera abundance between high (above median) and low (below median) fasting blood glucose in mice maintained on western diet determined by ANCOM analysis. Structural zero represents the presence of the bacteria in one group and the complete absence in another group. **(C–E)** Relative abundance of the *Akkermansia* in mice with high (above median) and low (below median) fasting blood glucose levels in mice maintained on the **(C)** KD, **(D)** MeD, and **(E)** WD. Mean abundance was compared using the Wilcoxon rank-sum test. For **(C–E)**, “***” = *p* < 0.001, and “ns” = not significant. **(F)** Differential genera abundance between high (above median) and low (below median) glucose metabolism parameters determined by ANCOM analysis. 20 most differentially abundant (based on cumulative ANCOM W-value) were selected for the graph. For easier presentation, ANCOM W values were converted to negative if CLR effect size is negative. Red indicates higher genera abundance in the above-median group and blue bacteria represents higher genera abundance in the below-median group. White represents a non-significant result obtained from ANCOM analysis. Models were adjusted for mouse strain and sex as confounding factors. ANCOM models were FDR (BH-method) corrected for multiple comparisons and a significant sub-hypothesis test at level adj.*P* < 0.05 was counted toward the W value. The median was calculated for each diet. For **(F)**, “***” = ≥W_0.9_, “**” = ≥W_0.8_, “*” = ≥W_0.7_, and “.” = ≥W_0.6_.

Glucose metabolism is an important part of the host metabolism and impaired glucose metabolism is directly related to metabolic diseases notably type 2 Diabetes Mellitus. Therefore, we then focused on the interaction between diet and microbiome on host glucose metabolism. Consistent with the host body composition, a significant diet ^*^ microbiota interaction affecting glucose metabolism was observed. For instance, more pronounced associations between fasting blood glucose, glucose tolerance, and microbial α-diversity were observed with WD compared to other diets in the study (Figure 4; Supplementary Table 11). Faith's PD explained about 16, 0.4, 2, 4, and 37% variability in GTT-AUC for mouse control chow, JD, KD, MeD, and WD, respectively, indicating that the WD altered-gut microbiota influences host glucose metabolism the most among all study diets. Similar to α-diversity, β-diversity measures also showed significant diet ^*^ microbiota interaction on host glucose metabolism (Figure 4A; Supplementary Table 12). For example, fasting blood glucose levels and glucose tolerance showed a significant correlation with β-diversity measures and the association was more prominent with WD, which was consistent with the α-diversity measures and body composition. The interaction remained significant even after adjusting for sex as a covariate in the ANOVA model (Supplementary Table 12). For instance, the interaction between weighted UniFrac PC1 and diet on GTT (as a dependent variable) remained significant (*P* = 0.03) after adjusting for sex. These results indicate that the overall microbial composition is associated with host glucose metabolism, but the association varies depending on the dietary pattern.

Next, we wanted to identify the specific bacterial ASV or genera responsible for modulating the host glucose metabolism in each of the study diets. Several gut bacterial genera (Figures 4B,F; Supplementary Table 13) and ASV (Supplementary Table 14) were found differentially abundant between high and low glucose metabolism parameters with each diet. For instance, *Akkermansia* was found associated with lower fasting blood glucose, glucose tolerance, and insulin levels, but only with WD (Figures 4C–E). *Faecalibaculum*, an SCFA producing bacteria, was associated with improved glucose tolerance only with MeD. Conversely, *Coriobacteriaceae UCG-002* was enriched in mice having higher fasting blood glucose levels compared to those having lower blood glucose levels with MeD, KD, and JD, but not with WD or controlled mouse chow. Unexpectedly, *Bifidobacterium* was positively associated with fasting blood glucose levels with MeD. Similar to the genus level, ASV level analysis also revealed significant diet ^*^ microbiota interactions on host glucose metabolism (Supplementary Table 14). For instance, an ASV belonging to the genus *Akkermansia* was found enriched in mice having lower fasting blood glucose and improved glucose tolerance, but only with WD. These results further support that diet ^*^ microbiota interaction modulates host glucose metabolism.

### Host genetics and gut microbiota interact to affect body composition

**Figure 5:**
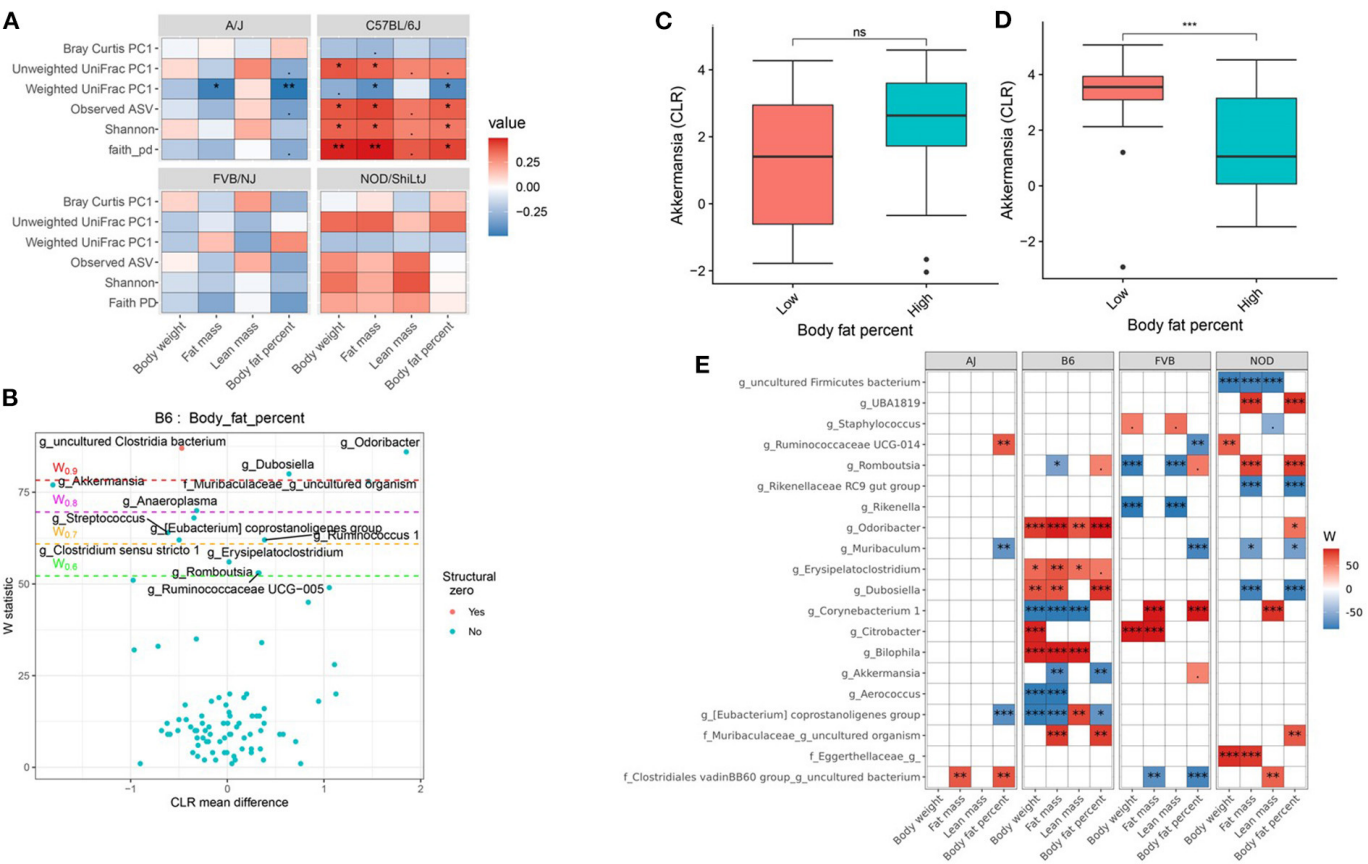
Host genetics and gut microbiota interact to modulate host body composition. **(A)** Association between body composition measures and α-diversity or first principal component (PC1) of β-diversity measured by mouse strains determined by using Spearman correlation. As indicated, red represents a positive correlation and blue represents a negative correlation. “***” = adj.*p* < 0.001, “**” = adj.*p* < 0.01, “*” = adj.*p* < 0.05, “.” = adj.*p* < 0.10, and “ns” = not significant. **(B)** Volcano plot of the differential genera abundance between C57BL/6J mice with high (above median) and low (below median) body fat percentage determined by ANCOM analysis. Structural zero represents the presence of the bacteria in one group and complete absence in another group. **(C)** Relative abundance of the *Akkermansia* in A/J mice with high and low body fat percentage. **(D)** Relative abundance of the *Akkermansia* in C57BL/6J mice with high and low body fat percentage. Mean abundance was compared using the Wilcoxon rank-sum test. For **(C,D)**, “***” = *P* < 0.001 and “ns” = not significant. **(E)** Differential genera abundance between high (above median) and low (below median) body composition measures determined by ANCOM analysis. 20 most differentially abundant (based on cumulative ANCOM W-value) were selected for the graph. For easier presentation, ANCOM W values were converted to negative if CLR effect size is negative. Red indicates higher genera abundance in the above-median group and blue bacteria represents higher genera abundance in the below-median group. White represents a non-significant result obtained from ANCOM analysis. Models were adjusted for diet and sex as confounding factors. ANCOM models were FDR (BH-method) corrected for multiple comparisons and a significant sub-hypothesis test at a level of adj.*P* < 0.05 was counted toward W-value. The median was calculated for each mouse strain. For **(E)**, “***” = ≥W_0.9_, “**” = ≥W_0.8_, “*” = ≥W_0.7_, and “.” = ≥W_0.6_.

Genetics is a critical determinate of disease risk and the interindividual heterogeneous response to diet. Thus, we next sought to determine if gut microbiota interacts with host genetics to affect clinical traits such as body composition. To evaluate this hypothesis, next, we compared the association between gut microbiota and host body composition within each mouse strain. The microbial diversity correlated significantly with host body composition and the association showed significant interaction with mouse strains, with a more pronounced association in C57BL/6J mice (Figure 5A; Supplementary Tables 15, 16). For example, body fat percentage was significantly correlated with all three α-diversity measures only in C57BL/6J (Figure 5A). In particular, Faith's PD showed the strongest correlation with body fat percentage, which explained 9, 19, 13, and 0.2% of the variability of the body fat percentage, for A/J, C57BL/6J, FVB/NJ, and NOD/ShiLtJ mice (Table 2), respectively, indicating that C57BL/6J is the most susceptible to gut microbiome aletration. Similarly, Shannon diversity index, observed ASV, Bray-Curtis, and unweighted UniFrac also explained higher percent variance of body composition in C57BL/6J (Table 2), further supporting that C57BL/6J mice are more susceptible to obesity due to diet-induced alteration of microbiota compared to other strains in this study.

**Table 2:** Percent phenotypic variance explained by gut microbial alpha and beta diversity measures in each of the mouse strains.

	**Faith's PD**	**Observed ASV**	**Shannon**	**Weighted UniFrac PC1**	**Unweighted UniFrac PC1**	**Bray Curtis PC1**
**A/J**
Body weight	3.3%	1.0%	0.9%	4.5%	0.8%	0.1%
Fat mass	6.5%	6.5%	0.2%	21.7%	3.0%	0.1%
Lean mass	0.0%	1.0%	3.7%	0.5%	8.1%	0.7%
Body fat percentage	9.3%	11.9%	3.6%	24.3%	9.2%	1.6%
Fasting glucose	1.0%	4.1%	14.9%	8.7%	27.2%	10.1%
Insulin	0.0%	0.5%	27.3%	0.6%	28.6%	22.7%
GTT	5.6%	4.6%	5.3%	0.0%	6.2%	4.4%
**C57BL/6J**
Body weight	23.4%	16.1%	14.1%	8.5%	16.0%	4.8%
Fat mass	24.3%	18.4%	14.7%	16.0%	13.7%	6.6%
Lean mass	15.2%	10.5%	8.7%	0.5%	8.6%	2.2%
Body fat percentage	18.8%	14.3%	10.5%	18.1%	9.4%	30.0%
Fasting glucose	39.3%	34.7%	30.2%	11.4%	32.1%	15.7%
Insulin	40.1%	32.7%	42.0%	25.7%	34.9%	25.4%
GTT	2.0%	0.1%	5.0%	0.6%	3.8%	9.9%
**FVB/NJ**
Body weight	2.5%	0.1%	1.3%	3.8%	3.6%	1.1%
Fat mass	10.1%	3.9%	3.3%	2.3%	0.6%	2.0%
Lean mass	0.1%	3.8%	0.1%	9.8%	6.4%	5.7%
Body fat percentage	13.3%	9.3%	5.9%	7.5%	0.1%	7.3%
Fasting glucose	2.0%	7.9%	0.5%	12.4%	4.6%	14.1%
Insulin	1.6%	4.8%	2.6%	47.1%	35.9%	21.4%
GTT	1.1%	7.7%	1.3%	3.8%	0.9%	4.3%
**NOD/ShiLtJ**
Body weight	4.9%	7.6%	11.4%	4.8%	12.5%	0.1%
Fat mass	3.3%	3.0%	4.2%	4.4%	13.6%	0.4%
Lean mass	6.5%	12.0%	16.1%	2.5%	2.3%	2.4%
Body fat percentage	0.2%	0.0%	0.0%	3.1%	11.8%	2.0%
Fasting glucose	1.1%	4.0%	0.0%	4.8%	17.9%	4.0%
Insulin	0.0%	0.0%	0.0%	0.0%	0.0%	0.0%
GTT	1.7%	0.6%	13.6%	5.6%	26.4%	3.5%

Percent variance explained was calculated from the correlation coefficient.

Then to detect the individual gut bacterium contributing to the host body composition, we determined that the differential abundance of genera (Figures 5B,E; Supplementary Table 17) and ASV (Supplementary Table 18) between high and low body weight, fat mass, lean mass, and body fat percentage in each of the mouse strains. For instance, *Akkermansia* was negatively associated with body fat percentage in C57BL/6J mice (Figure 5D), but not in other mice including A/J (Figure 5C). On the other hand, *Muribaculum* was enriched in A/J, FVB/NJ, and NOD/ShiLtJ mice with lower body fat percentage compared to those who had higher body fat percentage, which was not observed in C57BL/6J mice (Figure 5E; Supplementary Table 17). Similarly, *Ruminococcaceae UCG-014* was enriched in FVB/NJ mice with lower body fat percentage compared to mice having higher body fat percentage, but was not found in other mice. Detailed statistics of the distribution of the gut microbial genera and ASV between low and high groups have been depicted in Supplementary Tables 17, 18. Our results demonstrated that depending on the host genetics, the same gut bacteria may have a different association with body composition.

### Host genetics and gut microbiota interact to affect host glucose metabolism

**Figure 6:**
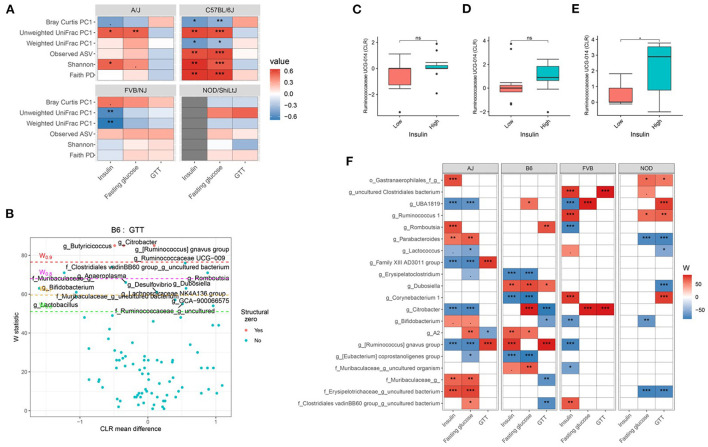
Host genetics and gut microbiota interact to modulate host glucose metabolism. **(A)** Association between host glucose metabolism phenotypes and α-diversity or first principal component (PC1) of β-diversity measured by mouse strains determined by using Spearman correlation. As indicated, red represents a positive correlation and blue represents a negative correlation. “***” = adj.*p* < 0.001, “**” = adj.*p* < 0.01, “*” = adj.*p* < 0.05, “.” = adj.*p* < 0.10. **(B)** Volcano plot of the differential genera abundance between C57BL/6J mice with high (above median) and low (below median) GTT AUC determined by ANCOM analysis. Structural zero represents the presence of the bacteria in one group and the complete absence in another group. Relative abundance of the *Ruminococcaceae UCG-014* in AJ **(C)** C57BL/6J **(D)** and FVB/NJ **(E)** mice with high and low plasma insulin levels. Mean abundance was compared using the Wilcoxon rank-sum test. For **(C–E)**, “*” = *P* < 0.05 and “ns” = not significant. **(F)** Differential genera abundance between high (above median) and low (below median) blood glucose-related phenotypes determined by ANCOM analysis. 20 most differentially abundant (based on cumulative ANCOM W-value) were selected for the graph. For easier presentation, ANCOM W values were converted to negative if CLR effect size is negative. Red indicates higher genera abundance in the above-median group and blue bacteria represents higher genera abundance in the below-median group. White represents a non-significant result obtained from ANCOM analysis. Red and blue represent significant associations determined by ANCOM. Models were adjusted for diet and sex as confounding factors. The median was calculated for each mouse strain. ANCOM models were FDR (BH-method) corrected for multiple comparisons and a significant sub-hypothesis test at a level of adj.P < 0.05 were counted toward W value. For **(E)**, “***” = ≥W_0.9_, “**” = ≥W_0.8_, “*” = ≥W_0.7_, and “.” = ≥W_0.6_.

Consistent with body composition data, a host genetics ^*^ gut microbiota interaction was also observed on the host glucose metabolism phenotypes (Figure 6A; Supplementary Tables 19, 20). For instance, in C57BL/6J mice, microbial α-diversity and fasting blood glucose levels were highly correlated, but not in FVB/NJ and NOD/ShiLtJ mice (Figure 6A). The associations between glucose metabolism phenotypes and β-diversity measures were also more pronounced in C57BL/6J mice (Figure 6A) indicating the overall composition of the microbial community is associated with altered metabolic health, which depends on the host genetics. Shannon diversity index explained 15, 30, 0.5, 0.03% of the variance for fasting blood glucose in A/J, C57BL/6J, FVB/NJ and NOD/ShiLtJ mice, respectively, indicating that the C57BL/6J strain is the most susceptible to metabolic diseases due to alteration in gut microbiota (Table 2).

Next, we focused on the specific gut bacteria that might be responsible for the elevated glucose phenotypes for each mouse strain and found that several gut bacterial genera (Figures 6B–F; Supplementary Table 21) and ASV (Supplementary Table 22) were differentially abundant between mice with high and low blood glucose, GTT, and insulin levels. In particular, C57BL/6J mice with lower GTT-AUC had significant enrichment of several gut bacterial genera including *Bifidobacterium, Lactobacillus*, and a number of genera under Muribaculaceae family lineage compared to those who had higher GTT-AUC (Figures 6B,F; Supplementary Table 21). Both the differential ASV and genera abundances reconfirmed the existence of significant host gene-microbiota interactions on glucose metabolism in the host. For instance, a higher abundance of *Ruminococcaceae UCG-014* was associated with higher insulin concentration in FVB/NJ (Figure 6E) mice but not in A/J mice (Figure 6C) mice. A higher abundance of *Ruminococcaceae UCG-014* in C57BL/6J was also associated with elevated insulin levels but was not significant (Figure 6D). Our results indicate that both genetics and gut microbiota play a role in an individual's glucose metabolism.

## Discussion

Metabolic syndrome is a major global public health problem (6, 8), which carries a huge health care cost burden (7). It is well accepted that diet and nutrition play a central role in the development of metabolic diseases (59). In particular, a WD pattern contributes to the global epidemic of metabolic diseases (9, 60). Additionally, host genetics contributes to the susceptibility to diet-induced metabolic diseases (22, 61). Similar to the host's genome, gut microbiota, also known as a malleable third genome (62), plays an important role in metabolic diseases (23, 63). Therefore, it is important to not only study how genetics, diet, and gut microbiota affect metabolism and disease risk but also to take an integrative approach to understand how interactions among these factors affect an individual's metabolic health. Such an approach can further enrich our understanding of the underlying mechanism for the interindividual variation in susceptibility to metabolic diseases. Our studies presented here are among several that have begun to model these interactions using mice (36, 64).

In this study, we treated four inbred mouse strains (A/J, C57BL/6J, FVB/NJ, and NOD/ShiLtJ) with four diverse diet patterns (Mediterranean diet, Japanese diet, ketogenic diet, Western diet), and a control mouse chow for 24 weeks starting from 6 weeks of age (22). We carefully selected four different inbred strains based on their phenotypic diversity and wider utilization within biomedical research, and the four diets were selected based on their effects on metabolic health. We previously reported that genetics is an important modulator of the diet effect on metabolic health (22) and here, we focus on perturbations to the gut microbiota and its interactions with diet, and host genetics to alter host metabolic health. Though several studies have evaluated diet and microbial effect on host metabolism, largely in C57BL/6J mice, the holistic approach that has been taken in this study using four different mouse strains and five diets is a major strength of our study. We report several key findings in this article including: (1) effect of popular human diets (MeD, KD, and JD) on gut microbiota in metabolically distinct inbred mouse strains; (2) gut microbial modulation of diet effect on host body composition and glucose metabolism; and (3) influence of host genetics on the association between microbiota and metabolic health. Each of these is discussed below.

### Effect of popular human diets on gut microbiota

Analysis of the gut microbial diversity and composition suggests that diet has a stronger influence on gut microbiota than host genetics, which is consistent with a previous report (64). We observed several notable diet effects on microbial community structure which we briefly described. Consistent with other studies (65), we observed that the microbial α-diversity increased in MeD groups compared to both WD and control mouse chow. MeD contains higher fruits, vegetables, whole-grain cereals, antioxidants, and polyphenols that have been hypothesized to support the survival and colonial expansion of diverse gut bacteria (65). Similarly, we observed that KD, which contains almost no carbohydrate, decreased the microbial α-diversity significantly across all mouse strains. Other studies have also found a reduction of gut microbial α-diversity of gut microbiota when mice are fed KD (66, 67). Since the large portion of the gut microbiota predominantly depends on undigested carbohydrates (68), it is expected that the very low carbohydrate in KD will decrease the microbial diversity. Additionally, the KD contains only a few, less diverse sets of ingredients (22), which also may cause the reduction of microbial diversity. The effects of a KD may depend on genetics or disease state as the NOD/ShiLtJ mice did not have reduced diversity due to KD (also to other diets in the study). Usually, NOD/ShiLtJ develops diabetes at 10–14 weeks (69). Our NOD/ShiLtJ mice were 30 weeks old, and their insulin levels were nearly undetectable, indicating the development of severe insulin deficiency in these mice. One possibility is that the microbiota composition in NOD/ShiLtJ is determined by diabetic phenotype, which is well known to affect microbial diversity (27). Contrary to our hypothesis that a JD would dramatically affect α-diversity, the JD and WD-fed mice had similar α-diversity. Among the four popular diets in this study, JD was studied the least and we did not find any previous study that compared classical JD with WD. However, one study found that the α-diversity of gut microbiota in individuals with habitual JD with higher JD score is not different than those who had a westernized low JD score diet (70), which supports that the JD may result in a microbial diversity similar to WD. However, JD has been found associated with better metabolic health compared to WD (22).

Diversity metrics are important, but the underlying composition of the gut microbiota (i.e., the specific microbes) also is important. Therefore, to understand the underlying microbial compositional differences between study diets, we next determined differential gut bacteria abundance in KD, JD, and MeD compared to WD. Consistent with the nutrient diversity, the underlying composition of gut bacteria was quite different among the study diets. For example, we found that several gut bacteria abundances including *Lactococcus, Streptococcus*, and *Parabacteroides* were higher in JD compared to WD, whereas, relative abundance of *Odoribacter, Rikenella*, and *Staphylococcus* were lower in JD compared to WD. Similarly, a genus under the family *Muribaculaceae* [previously referred to as *S24-7* (71)] was enriched over four-fold in the gut microbiota of WD compared to JD in all mouse strains. These data suggest that a closer look at the underlying composition of bacteria in combination with microbial diversity is needed to completely understand the health effects of gut microbiota.

### Gut microbiota modulate diet effect on host health

Beyond the regulatory effects that both diet and genetics have on the composition of the microbiota, it is important to understand the functional differences that altered gut microbiota have on clinical or disease related traits. Of particular interest are traits related to metabolic diseases. We highlight 2 specific examples from the current study. Determining the exact mechanisms for the effect of specific microbiota/diet interactions is difficult but evolving understanding of the metabolic consequences of specific gut bacteria does provide some clues. For example, we found that *Akkermansia* is associated with lower fasting blood glucose levels and glucose tolerance and insulin levels. *Akkermansia* is an anaerobic, intestinal mucin-degrading, a gut bacterium (72) uses mucin, has been found lower in diabetic patients (27) and is being considered as a potential probiotic for treating metabolic diseases (73). However, our study reveals that the inverse relationship between *Akkermansia* and diabetic phenotypes varies among the diets and was only observed significantly in WD-treated mice suggesting that the health effects of specific microbes may be found only within the context of a specific diet pattern. One possible explanation is that WD induces metabolic inflammation (2) and *Akkermansia* and its membrane protein Amuc 1100 reduces inflammation through Toll-like receptor 2 (74). In addition to direct host cell signaling (ie- Amuc1100 and TLR2) there has been considerable interest in the local metabolic products produced by specific microbes and how they affect health. We identified a higher abundance of *Faecalibaculum* in MeD fed mice is significantly associated with a lower glucose tolerance which is consistent with literature suggesting that adherence to MeD reduces the risk of developing type 2 diabetes (75). *Faecalibaculum* is known to be an efficient SCFA producer in the rodent gut bacterium (76), and higher concentrations SCFA in stool improves metabolic health (27). Our study results indicate that enrichment of some gut bacteria (e.g., *Faecalibaculum*) and perhaps their ability to produce metabolites beneficial to the host might be responsible (at least partly) for some of the improved health outcomes ascribed to diet. Several studies have reported that *Bifidobacterium* improves metabolic health in humans (27, 77). Unexpectedly, we found *Bifidobacterium* to be positively associated with fasting blood glucose levels. Two important concepts could account for this surprising result. *Bifidobacterium* is a genus composed of 54 different species identified as of January 2017 (78), and not all *Bifidobacterium* are equal. Many of these species are animal host-specific, with evidence of vertical transmission from mothers to offspring (78, 79). Among different species of the *Bifidobacterium, Bifidobacterium Longum* subsp. *infantis i*s the most beneficial to health (80). Some of the species in *Bifidobacterium* lineage including *Bifidobacterium bifidum* do not have this beneficial effect. With the 16S rRNA gene amplicon sequencing data, we only can identify genus level taxonomical information. *Bifidobacterium longum* subsp. *infantis* utilizes human milk oligosaccharides and is thus predominantly found in breastfed, human infants (81). *Bifidobacterium longum* subsp. *infantis* does not colonize in mice (82) which confounds *Bifidobacterium* comparisons across human and mouse studies. Thus, the positive association between *Bifidobacterium* and increased blood glucose levels is most likely not driven by *Bifidobacterium longum* subsp. *infantis*. Utilization of metagenomics is needed to completely understand the association between *Bifidobacterium* and carbohydrate metabolism in mice.

### Influence of host genetics

The diversity and composition of the gut microbiota depend on several factors including the host's digestion, metabolism, and intestinal immunity (83, 84), all of which are influenced by host genetics (22, 85, 86). Therefore, it is expected that changes in gut microbiota due to diet will be different among individuals depending on the host genetics. The effect of dietary patterns on gut microbial α-diversity, β-diversity, and composition was heterogeneous and varied in effect size between mouse strains utilized in this study. This supports the notion that genetics is a critical underlying mediator of the diet-microbiome axis. For example, the NOD/ShiLtJ mice did not have reduced diversity due to KD (also to other diets in the study). Usually, NOD/ShiLtJ develops diabetes at 10–14 weeks (69). Our NOD/ShiLtJ mice were 30 weeks old, and their insulin levels were nearly undetectable, indicating the development of severe insulin deficiency in these mice. One possibility is that the microbiota composition in NOD/ShiLtJ is determined by diabetic phenotype which is well known to affect microbial diversity (27). In addition to diversity, we also observed several differential bacterial abundances that vary depending on host genetics. For instance, *Roseburia* abundance in NOD/ShiLtJ mice was significantly increased in the KD and JD compared to WD but decreased in the MeD. Our study finding of the interaction between diet and host genetics on gut microbiota is in line with a few published reports. For example, a study (87) reported an interaction between diet and host genetics on microbiome using two inbred mice and two experimental diets. Similarly, another study has reported that different mouse strains have a different distribution of gut microbiota and enterotypes in C57BL/6J, FVB/NJ, BALB/cJ, NOD/ShiLtJ, and an outbred Swiss mouse strain (88). A recent study (89) showed that the alteration of gut microbial diversity and composition due to fructose consumption is different among C57BL/6J, DBA/2J, and FVB/NJ mice strains, indicating diet ^*^ genetic interaction on gut microbiota similar to the current study.

Mice and humans share a similar genetic architecture which makes mice an efficient model for genetic research. The ability to control environmental exposures including diet makes it a widely used animal model for studying human health and diseases (90). Therefore, to better understand the influence of genetics on metabolic health, we determined the interactions between mouse strain and gut microbiota on host body composition and carbohydrate metabolism. As mentioned earlier, different mouse strains have different metabolic profiles (22, 85, 91). Therefore, it is expected that host metabolic response to the gut microbiota and microbial metabolites will be different among the mouse strains because of their differences in genetic background. In our study, we found significant genetics ^*^ microbiota interaction on host body composition and metabolic health. Consistent with our findings, a recent study (89) determined that the correlations between host fructose-responsive genes expression, adiposity and gut microbial composition are modulated by host genetics. Additionally, they also found that gut microbiota in C57BL/6J is more likely to be correlated with adiposity compared to DBA/2J and FVB/NJ mice. We also found that the C57BL/6J mice have the strongest correlation between gut microbiota and body composition. We did not find a set of bacteria consistently associated with body composition and metabolic phenotypes across all study diets and mouse strains. Similar to ours, a recent study (92) also did not find a set of common gut bacteria is associated with cancer immune therapy across 5 different human cohorts. Our findings indicate the importance of considering both diet and genetics for designing probiotics for precision therapeutics.

One limitation is that this study did not determine sex-specific effects due to insufficient mouse number in the female group for some diet-mouse strain arms. Therefore, our study result was presented here as a combined effect on both sexes upon adjusting for sex as a covariate in the statistical model. A future study with adequate power is needed to determine the interactions between phenotype, mouse strain, diet, and biological sex. We only collected stool samples at the end of the study to measure microbiota. Collecting more stool samples at multiple time points including a baseline would help us better understand the subtle and cumulative effect of diet on microbial composition and the dynamic association between microbiota and metabolic phenotype over time. Additionally, we did not perform a functional validation study with a fecal microbiota transplant (FMT) in this study, which would confirm the causality. More research with larger sample sizes is needed to determine the sex dimorphic nature of the associations between diet, microbiota, and genetics on metabolic health.

In conclusion, the present study demonstrates diverse diet patterns affect gut microbial community structure and that these can be influenced by host genetics. The interactions of diet and genetics likely contribute to the modulatory effects of diet plays in metabolic health. Importantly, depending on the composition of the gut microbiota and host genetics, the same diet can have dramatically different metabolic outcomes which is a consideration as we move toward “precision nutrition”.

## Data availability statement

The datasets presented in this study are deposited in the NCBI's SRA repository (https://www.ncbi.nlm.nih.gov/), accession number PRJNA773750.

## Ethics statement

The study protocol was approved by Texas A&M University Institution Animal Care and Use Committee (IACUC) protocol number 2017-0076. All experiments were performed in accordance with relevant guidelines and regulations.

## Author contributions

BB and DT designed the study, supervised all analyses, interpreted the results, and mentored manuscript writing. MH, AS, WB, CG, ED'S, and LD conducted the research. MH performed the bioinformatics of the gut microbiota and statistical analyses, interpreted the results, drafted the manuscript, and addressed co-authors comments and concerns. BB, DT, AS, WB, CG, ED'S, and LD critically revised the manuscript. BB has primary responsibility for final content. All authors read and approved the final manuscript.

## Funding

This research was supported in part by USDA project 2032-51530-025-00D (BB), National Institutes of Health (NIH) grant R01HL128572 (BB), CA-105417 (DT), and HG-008529 (DT). Phenotypes were collected using the Texas A&M Institute for Genome Sciences and Society Rodent Phenotyping Core, and the Animal Metabolism Phenotyping core facility within the University of Northern Carolina's Nutrition Obesity Research Center (NORC) (NIH grant DK-056350).

## Conflict of interest

The authors declare that the research was conducted in the absence of any commercial or financial relationships that could be construed as a potential conflict of interest.

## Publisher's note

All claims expressed in this article are solely those of the authors and do not necessarily represent those of their affiliated organizations, or those of the publisher, the editors and the reviewers. Any product that may be evaluated in this article, or claim that may be made by its manufacturer, is not guaranteed or endorsed by the publisher.
