# Supplementary material for: Gut microbiota and host genetics modulate the effect of diverse diet patterns on metabolic health
Source: Front Nutr. 2022 Aug 18;9:896348. doi: 10.3389/fnut.2022.896348 (PMC9434023; doi:10.3389/fnut.2022.896348)
Supplement: Supplementary file 2 [file Data_Sheet_2.docx]

**Supplemental information – Supplemental Figures**

**Manuscript title**: Both Host Genetics and Gut Microbiota Play a Role in Improving Metabolic Health Through Precision Nutrition in Mice

**Authors**: M. Nazmul Huda, Anna C. Salvador, William T. Barrington, C. Anthony Gacasan, Edeline M. D’Souza, Laura Deus Ramirez, David W. Threadgill, Brian J. Bennett^*^

**Supplemental Figures**

**Supplemental Figure 1: Study design**. A/J, C57BL/6J, FVB/NJ, and NOD/ShiLtJ male and female mice were maintained on the Mediterranean diet (MeD), Japanese diet (JD), traditional American diet (Western diet (WD)), Ketogenic diet (KD), or control mouse diet for 24 weeks. Anthropometric data or sample collection events were conducted as indicated by the corresponding diamond symbol in the figure. Gut microbiota data was available from n= 27-34 per diet, n=27-44 mice per strain, and n=3-10 per diet per strain.


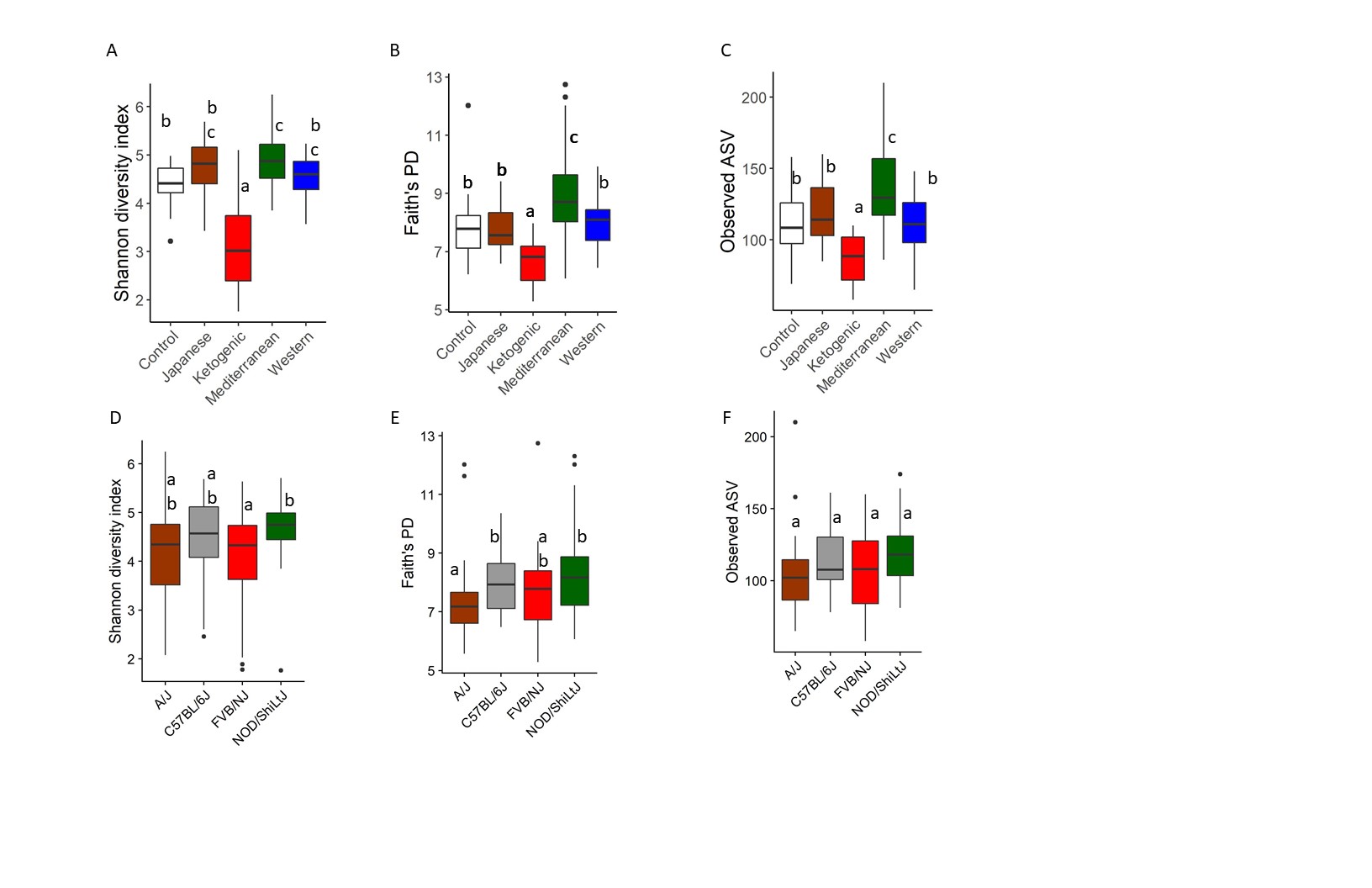


**Supplemental Figure 2**: Comparison of Shannon diversity index (**A** and **D**), Faith’s PD (**B** and **E**), and Observed ASV (**C** and **F**) among diet groups (first row) and mouse strains (second row). Boxes without a common letter are significantly (p < 0.05) different from others. Boxes with common letters represent no significant difference. ANOVA with Tukey's post hoc analysis was performed to determine the statistical differences.


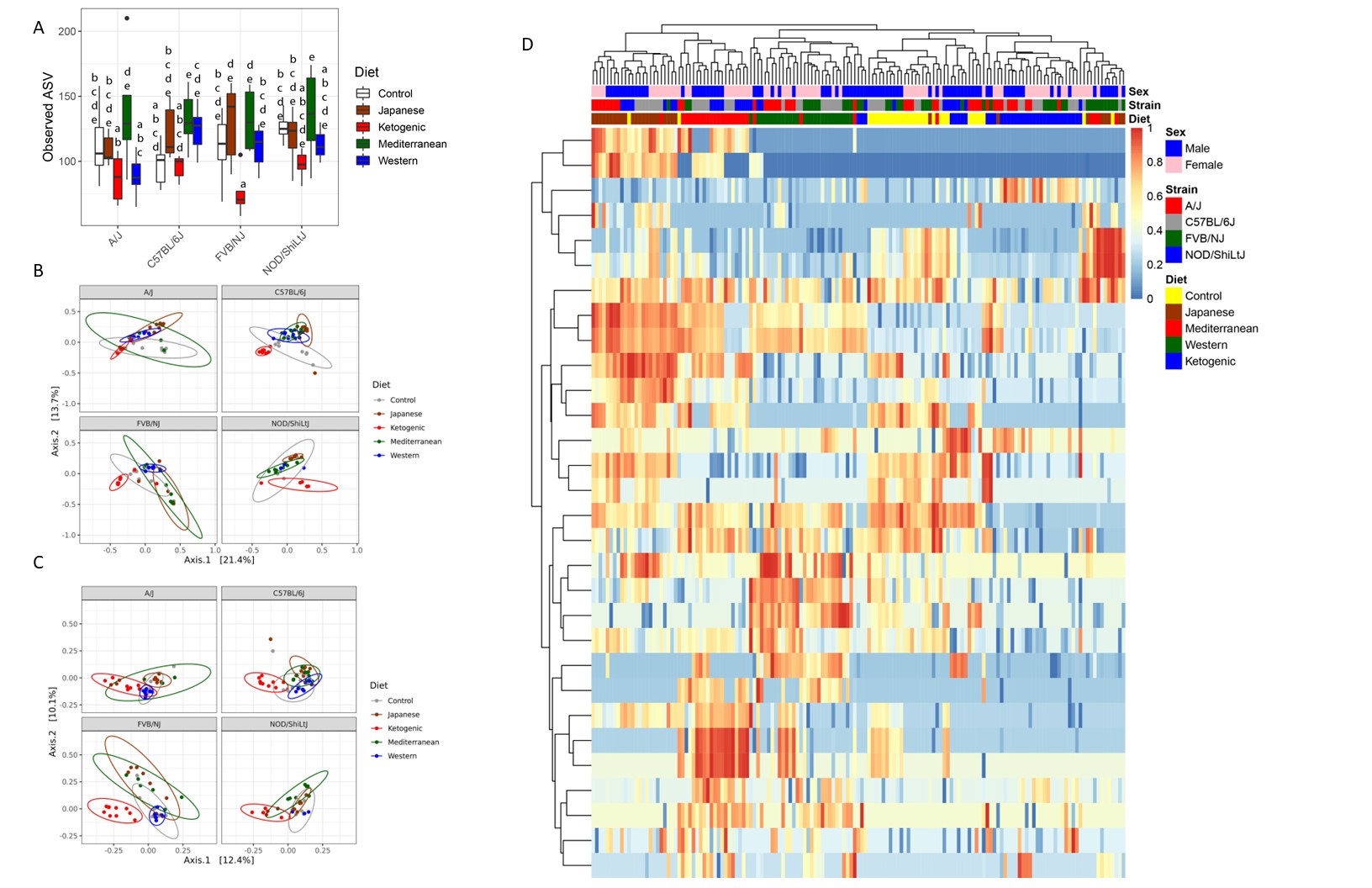


**Supplemental Figure 3**: (**A**) Observed species α-diversity by diet and strain. Boxes without a common letter are significantly (p<0.05) different from others. (**B**) Unweighted UniFrac, and (**C**) Bray-Curtis β-diversity PCoA plots. (**D**) Heatmap represents the relative abundance of top 30 differentially abundant bacterial ASV among diet determined by ANCOM-2.1. The X-axis represents the sample and Y-axis represents the bacterial ASV. Values were scaled from zero to one. ANCOM model was adjusted for sex.


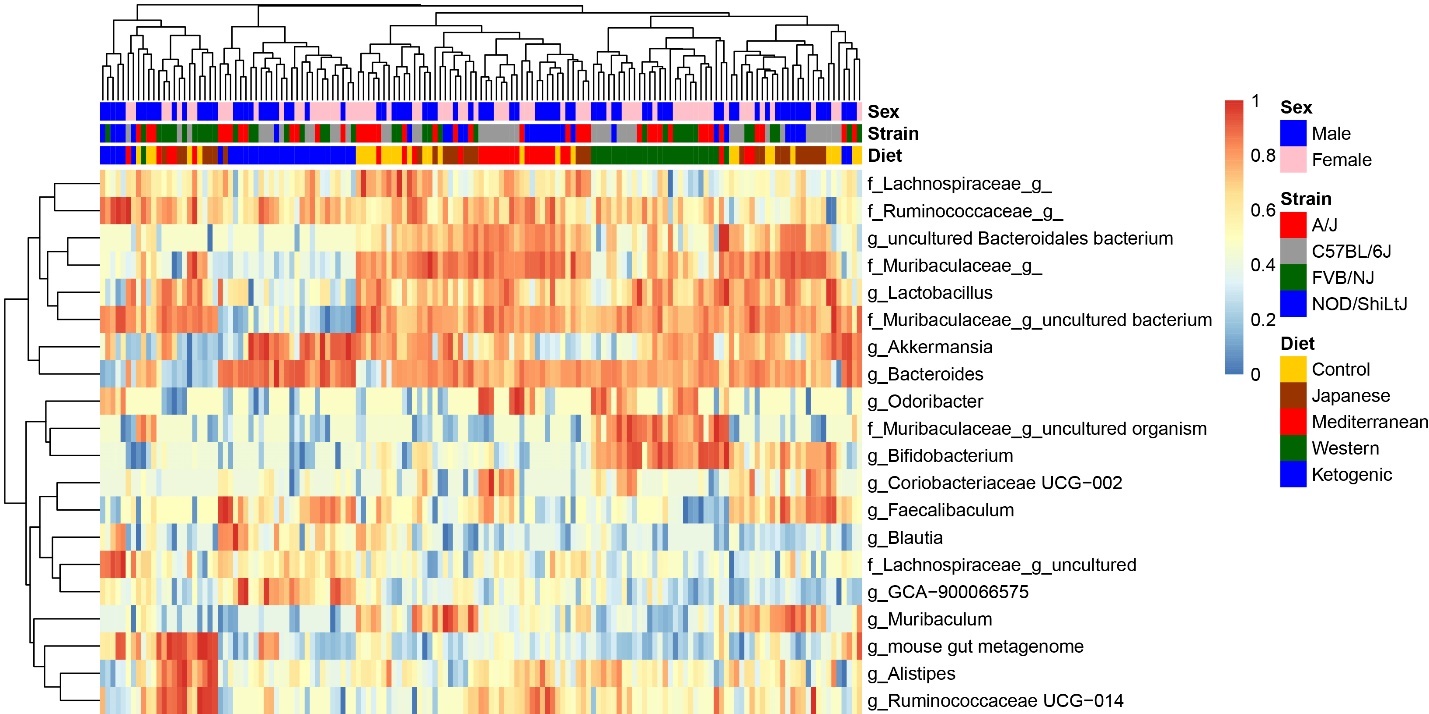


**Supplemental Figure 4**: Heatmap represents the relative abundance of the top 20 most abundant bacterial genera. The X-axis represents the sample and Y-axis represents the bacterial genera. Values were scaled from zero to one.


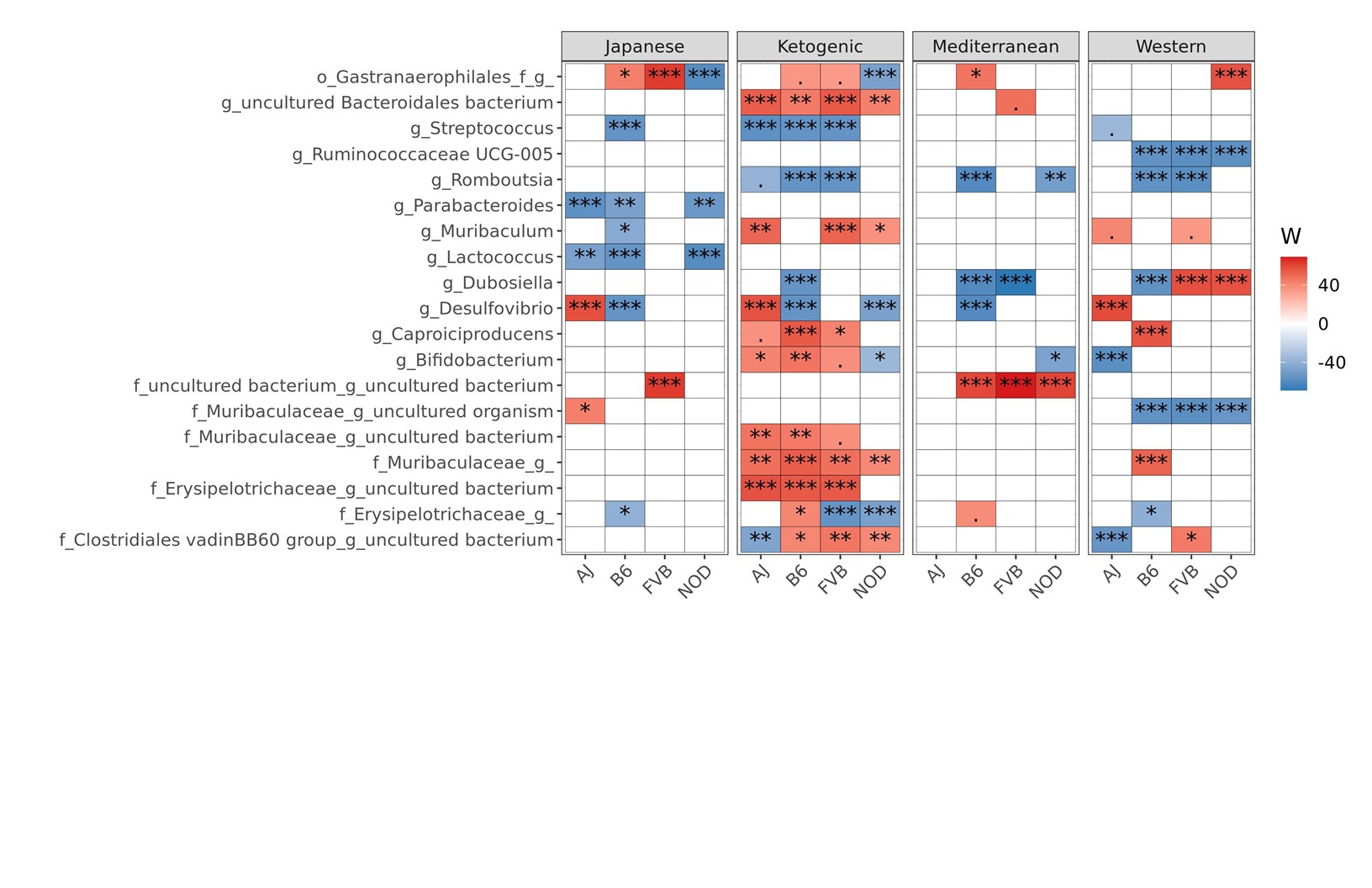


**Supplemental Figure 5**: **Differential genera abundance in Mediterranean, Ketogenic, Japanese or Western diets compared to mouse control diet.** Differential genera abundance in the Mediterranean, Ketogenic, Japanese, or Western diets compared to control mouse diet. The top 20 most influenced bacterial genera (based on cumulative ANCOM W value) were selected for the graph. For easier presentation, ANCOM W values were converted to negative. Red indicates a higher abundance of bacteria is lower in the Mediterranean, Ketogenic, or Japanese diets compared to the mouse control diet, whereas blue represents the opposite relationship. White represents a non-significant result obtained from ANCOM analysis. Red and blue represent significant associations determined by ANCOM after FDR correction for multiple comparisons at a significant level adj.P < 0.05. The full list of differential genera abundance with detailed statistics has been depicted in **Supplemental Table 4**. ANCOM models were adjusted for strain and sex as confounding factors.


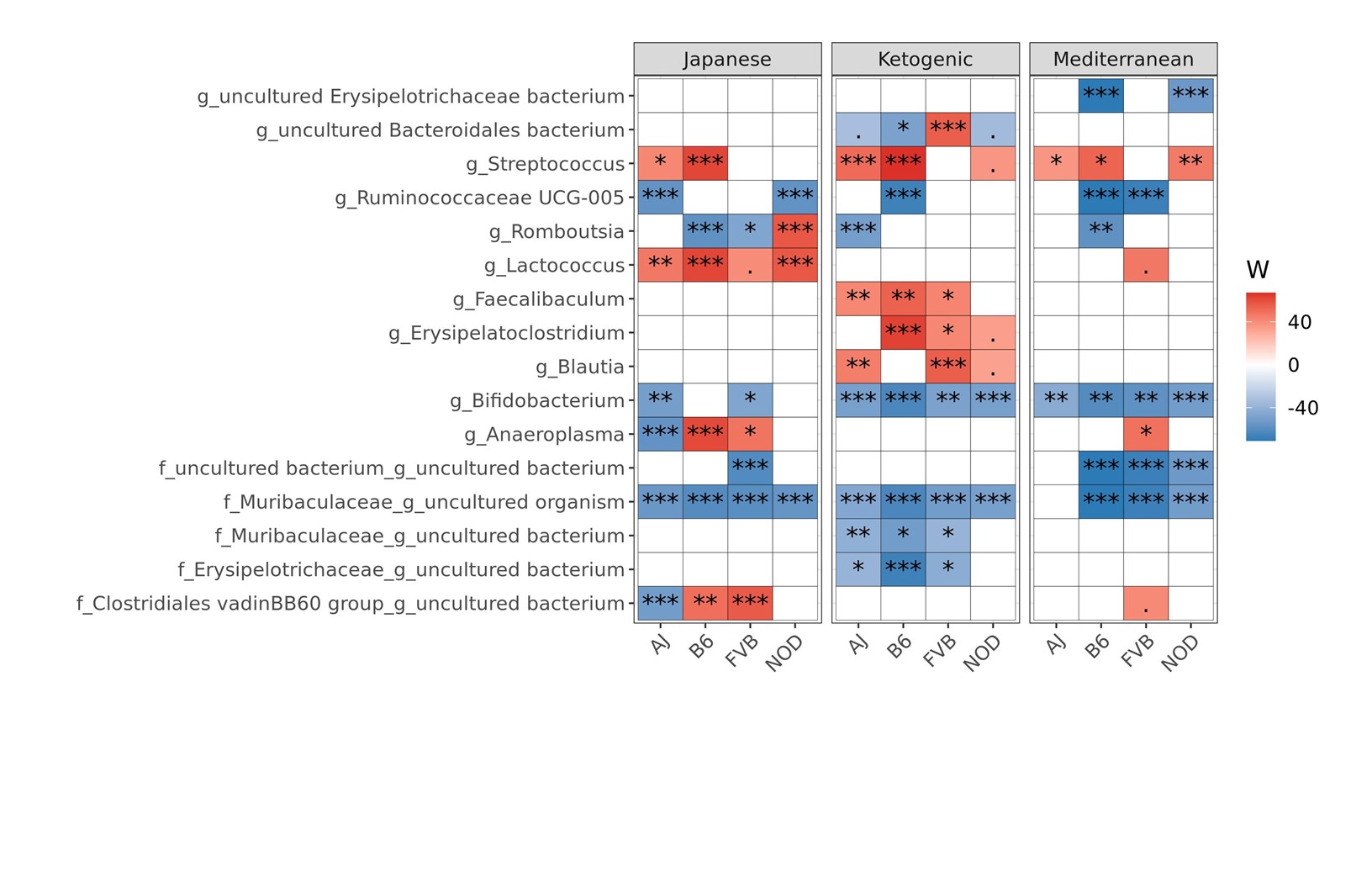


**Supplemental Figure 6**: **Differential genera abundance in Mediterranean, Ketogenic, or Japanese diets compared to Western diet.** Differential genera abundance in the Mediterranean, Ketogenic, and Japanese diets compared to Western diet. The top 20 most influenced bacterial genera (based on cumulative ANCOM W value) were selected for the graph. For easier presentation, ANCOM W values were converted to negative. Red indicates a higher abundance of bacteria is lower in the Mediterranean, Ketogenic, or Japanese diets compared to the mouse control diet, whereas blue represents the opposite relationship. White represents a non-significant result obtained from ANCOM analysis. Red and blue represent significant associations determined by ANCOM after FDR correction for multiple comparisons at a significant level adj.P < 0.05. The full list of differential genera abundance with detailed statistics has been depicted in **Supplemental Table 5**. ANCOM models were adjusted for strain and sex as confounding factors.
